# Can dietary supplementation with highly purified fucoidan alter growth, digestive enzyme activity, serum biochemicals, immune-antioxidant responses, and related gene expressions in Nile tilapia (*Oreochromis niloticus*)?

**DOI:** 10.1371/journal.pone.0339270

**Published:** 2026-07-10

**Authors:** Eman Y. Mohammady, Mohamed R. Soaudy, Mohamed A. Elashry, Ehab R. El-Haroun, Mohamed S. Hassaan

**Affiliations:** 1 Aquaculture Division, National Institute of Oceanography and Fisheries, NIOF, Cairo, Egypt; 2 Department of Animal Production, Fish Research Laboratory, Faculty of Agriculture at Moshtohor, Benha University, Egypt; 3 Animal Production Department, Faculty of Agriculture, Cairo University, Cairo, Egypt; 4 Department of Integrative Agriculture, College of Agriculture and Veterinary Medicine, United Arab Emirates University, Abu Dhabi, United Arab Emirates; Universiti Malaysia Kelantan, MALAYSIA

## Abstract

The present study investigates the impact of dietary fucoidan supplementation on growth performance, intestinal tract morphology, endogenous digestive enzymes, hematological parameters, serum biochemical indices of Nile tilapia (*Oreochromis niloticus*), oxidative biomarkers, and related gene expressions. Fish weighing 2.54 ± 0.12 g had randomly assigned them into five groups of equal size (20 fish each aquarium), with three replicates per group. The fish were fed for 70 days with five experimental diets formulated: T1: Control (fucoidan 0 mg kg^-1^); T2: 0.5 mg kg^-1^ fucoidan; T3: fucoidan 1.0 mg kg^-1^, T4: fucoidan 1.5 mg kg^-1^ and T5; fucoidan 2.0 mg kg^-1^. At the end of the experiment, growth indices, feed utilization, digestive enzyme activity, intestinal histomorphometric, hematological indices, serum biochemical indices, and antioxidant enzyme activities were significantly (P < 0.05) increased in the groups fed diets supplemented by fucoidan, with the superiority of fish fed 2 mg kg^-1^ compared to the basal diet. Additionally, a control diet has the highest ALT and AST compared to other diets supplemented with different fucoidan levels. Fish fed either 1.5 mg kg^-1^ or 2.0 mg kg^-1^ fucoidan recorded the higher (P < 0.05) hematological parameters as WBCs, RBCs, neutrophils, lymphocytes, monocytes, and eosinophils. A diet supplemented with 2 mg kg^-1^ diet fucoidan displayed the highest gene expression of the *inf-γ* and *il-1β*, while the heat shock protein 70 (*hsp70*) gene was down-regulated. Overall, our results highlight the efficacy of fucoidan in increasing growth performance, feed utilization, antioxidant enzyme activity, and gene expression. Therefore, it can be considered a promising feed additive for tilapia farming.

## Introduction

Intensive aquaculture causes stress to fish and increases susceptibility to many diseases and immune suppression [1–3]. Though pathogen infection and disease outbreak are the main challenges for aquaculture industry sustainability, resulting in greater losses [4–6]. The Use of antibiotics, vaccines, and other chemicals is currently used in varying degrees to control disease. However, using antibiotics has their drawbacks, including inhibiting the immune system of aquatic animals, environmental hazards, and food safety problems [7,8]. In addition, the World Health Organization (WHO) prevents the using of antibiotics in aquafeeds to avoid various negative impacts on the aquaculture industry as follows: i) accumulation of antibiotics in the fish body tissues [9]., ii) The risk of antibiotic resistance in pathogenic bacteria [10]., iii) water pollution with residual of antibiotics has a negative impact of human activities [9] and iv) transfer of resistance to human pathogenic bacteria [11]. Consequently, novel strategies and new techniques should be implemented to control pathogen infections [1,12–16]. One of the promising feed additives in aquafeeds that stimulate the immune system of fish are natural bioactive compounds, natural immunostimulants, prebiotics, and probiotics, which have evicted to be progressively relevant as an effective alternative for prophylactic treatment against disease outbreaks in aquaculture systems. Newly, aquacultural research has paid much attention to fucoidan due to its functional properties as a potential immunomodulator [15,17–23].

Fucoidan is a natural polysaccharide that is mostly found in the cell wall of brown seaweed and marine invertebrates [24–26]. Fucoidan is recognized with many biological properties, such as low toxicity and oral bioavailability. Moreover, fucoidan has functional properties such as anti-inflammatory, anti-oxidative stress, and treatment for cancer [27–29]. From the point of view of pharmacology, fucoidan has been shown to benefit many physiological and nutritional functions and has beneficial effects and important functions on fish health, e.g., it helps prevent infections caused by intestinal pathogens, modulates a normal immunological response, and isanti-inflammatory, anti-carcinogenic, and anti-oxidative [29]. Recently, dozens of researchers in aquaculture have highlighted the importance of including Fucoidan in aquafeeds as growth promoters and immunostimulants [29]. Inclusion of fucoidan at 1% enhanced the performance and intestinal topography of seabass [19]. Moreover, enrichment of the diets of different fish species with fucoidan proved its role as an immunostimulant to boost immune system response and their resistance to the pathogen microbes [30]. Furthermore, the inclusion of Fucoidan in shrimp diets enhances growth performance and related gene and immune expression of *P. monodon* [31]. Also, fucoidan has been found to affects hematology parameters, antioxidant status, and nonspecific immune responses in catfish [20]. However, to the authors’ knowledge, the detailed supplemental effects of fucoidan on aquatic species performances are still not yet documented. In this context, the present work was designed to study the dietary effects of fucoidan on the growth, feed utilization, endogenous enzymes activity, immune status, growth-related gene expression, and survival of Nile tilapia, *Oreochromis niloticus*.

## Materials and methods

### Ethics statement

This trial was carried out in strict accordance with the recommendations in the Guide for the National Institute of Oceanography and Fisheries (NIOF) Ethical Committee for the Care of Aquatic Animals. All experiments and sampling were performed in accordance with Committee on the Ethics of Animal Experiments of the NIOF (Protocol Number: NIOF-AQ4-F-25-R-038). All sampled animals were euthanized using buffered 3-aminobenzoic acid ethyl ester (MS 222, 100 mg/L, Sigma, St. Louis, MO) following standard procedures.

### Fish and feeding protocol

Nile tilapia *O. niloticus* were obtained from a private farm (Kafer El Sheikh Governorate, Egypt) and acclimatized for two weeks before being fed commercial diets containing 30% CP at a rate of 3% of total biomass three times a day at 9:30 a.m., 11:30 a.m., and 3:30 p.m. for two weeks. The feeding trial was conducted at the National Institute of Oceanography and Fisheries. Following acclimation, Nile tilapia with an initial body weight (2.54 ± 0.12 g) were divided into five groups with three replicates (15 aquariums) for 70 days. Each aquarium (150 L^3^) is randomly stocked with 20 fish, with approximately 20% of the water changed daily. The tested diets were provided for the experimental fish satiation three times daily. The amount of feed consumed by each fish over the course of the experiment was calculated and expressed as a total. During the 70- day feeding trial, the water quality parameters averaged (±SD): water temperature, 27.2 ± 0.8 ^°^C; dissolved oxygen, 5.7 ± 0.3 mg L^-1^; total ammonia, 0.20 ± 0.11 mg L^-1^; nitrite, 0.07 ± 0.03 mg L^-1^; total alkalinity, 169 ± 42 mg L^-1^; chlorides, 565 ± 152 mg L^-1^ and pH 8.6 ± 0.3. Water quality parameters were maintained within the recommended range of Nile tilapia according to [32].

### Diets formulation

A basal diet (316.35 g kg^-1^ crude protein) was formulated (Table 1) and supplemented with 0, 0.5, 1.0, 1.5, and 2.0 mg of fucoidan kg^-1^ diets. Using a pelleting hand noodle maker, all the components were thoroughly combined with the fucoidan before being formed into pellets with a diameter of 2 mm. Fucoidan and oil were added to the mixed ingredients. These pellets were then allowed to dry overnight at room temperature and then kept at 4°C. Gross energy was determined as reported by [33] and is shown in Table 1 along with the proximate analysis of the ingredients and diets as analyzed by the [34] method. Fucoidan extracted from the brown seaweed species, *Undaria pinnatifida* was purchased from Sigma-Aldrich 9072-19-9 (Buchs, Switzerland) purity is > 86.

**Table 1:** Formulation and proximate composition of basal diet (g/kg diet, dry matter).

Ingredient	g/kg
Fish meal	100
Soybean meal	350
Corn gluten meal	30
Yellow corn	250
Wheat bran	100
Rice polishing	100
Fish oil	40
Premix	25
Vitamin C	5
Sericite	0
Chemical analysis %	
Protein	316.35
Lipid	70.30
Ash	56.69
Fiber	54.00
Nitrogen free extract^2^	502.66
Gross energy ^3^ MJ /kg	19.38

^1^Vitamin and mineral mixture kg^-1^ of mixture contains: 4800 I.U. Vit A, 2400 IU cholecalciferol (vit. D), 40 g Vit E, 8 g Vit K, 4.0 g Vit B_12_, 4.0 g Vit B_2_, 6 g Vit B_6_, 4.0 g, Pantothenic acid, 8.0 g Nicotinic acid, 400 mg Folic acid, 20 mg Biotin, 200 gm Choline, 4 g Copper, 0.4 g Iodine, 12 g Iron, 22 g Manganese, 22 g Zinc, 0.04 g Selenium. folic acid, 1.2 mg; niacin, 12 mg; d-calcium pantothenate, 26 mg; pyridoxine. HCl, 6 mg; riboflavin, 7.2 mg; thiamin. HCl, 1.2 mg; sodium chloride (NaCl, 39% Na, 61% Cl), 3077 mg; ferrous sulfate (FeSO_4_.7H_2_O, 20% Fe), 65 mg; manganese sulfate (MnSO_4_, 36% Mn), 89 mg; zinc sulfate (ZnSO_4_.7H_2_O, 40% Zn), 150 mg; copper sulfate (CuSO_4_.5H_2_O, 25% Cu), 28 mg; potassium iodide (KI, 24% K, 76% I), ^2^NFE (Nitrogen free extract) =100-(crude protein + lipid + ash +fibre content). ^3^Gross energy calculated using gross calorific values of 23.63, 39.52 and 17.15 kjg^-1^ for protein, fat and carbohydrate, respectively according to Brett (1973).

### Growth parameters

At the beginning and conclusion of the trial, growth parameter values were recorded; the equations used to calculate these values are as follows:

- WG = final weight (g) – initial weight (g)
- Specific growth rate (SGR) = LnW2 – LnW1/t (days), where, Ln = the natural log; W1 = initial fish weight, W2 = the final fish weight in grams and t = Period in days
- Feed conversion ratio (FCR) was calculated according to by the equation
- FCR = Feed intake (g)/weight gain (g)
- Protein efficiency ratio (PER) = Weight gain (g)/protein ingested (g).

### Digestive enzymes activity

Intestines from four fish in each aquarium of treatments were sampled, immediately rinsed with ice‐cold physiological saline, and then homogenized in 10 volumes (w/v) of the same saline solution and centrifuged at 5,000 *g* for 15 min at 4°C; then, the supernatant was stored for endogenous enzyme activity analysis [35]. Fish was fasted 24 hours before sampling. Chymotrypsin activity was estimated using the method of [36] with N‐benzoyl‐Ltyrosine ethyl ester (BTEE) as substrate. The diluted sample solution was added to 6 ml of 0.0005 M BTEE in Tris buffer (10.55 g CaCl_2_. 2H_2_O dissolved in 250 ml 0.2 M Tris [hydroxymethyl] aminomethane, adjusted to pH 7.8, diluted to 1 L, and 432 ml methanol added) and assayed at 254 nm. Trypsin activity was also measured according to [36] with Na‐p‐toluenesulfonyl‐L‐arginine methyl ester (TAME) as substrate at 247 nm. Following dilution, a 0.2 ml sample solution was added to 6 ml of 0.00104 M TAME in Tris buffer (1.47 g CaCl_2_. 2H_2_O dissolved in 200 ml 0.2 M Tris [hydroxymethyl] aminomethane diluted to 1 L, pH 8.1). Lipase activity was determined as described by [37]. The titration method was validated by using olive oil‐gum as the standard. Amylase activity was estimated according to [38], using starch as the substrate. For each assay, 1 ml of diluted sample was incubated for 3 min with 1% starch (1 g soluble starch and 0.035 g NaCl in 100 ml 0.02 M Na_3_PO_4_, pH 6.9). After 3 min, the reaction was stopped by the addition of 2 ml 3,5‐dinitrosalicylic acid reagent. The solution was then heated for 5 min in boiling water and then cooled with 20 ml distilled water added and then measured at 540 nm. Intestinal alkaline phosphatase activity was determined by the methods of [39] with 4-nitrophenyl phosphate as substrate at 405 nm.

### Intestinal histomorphometry

At the end of the feeding trial, three fish from each aquarium were anesthetized with 3-aminobenzoic acid ethyl ester (MS 222, 100 mg/L, Sigma, St. Louis, MO), dissected, and intestine samples were randomly taken. Afterwards, samples were washed in phosphate buffer saline (PBS), fixed in 10% formalin for 24 h, dehydrated in ascending grades of alcohol, and cleared in xylene. Then, samples were embedded in paraffin wax (congealing point 58–60 °C). The longitudinal and transverse sections, each of 6 μm thickness, were cut by using a Rotary Microtome (Reichert Technologies, NY, USA) and stained in hematoxylin and eosin (H&E) according to the standard procedure [40]. The tissue sections were examined under a light microscope equipped with a full HD microscopic camera and image analysis software (Leica Microsystem, Germany). The mean villus height (measured from the base to the top) was measured by image analysis software for statistical analysis [41].

### Hemato-biochemical analysis

At the end of the experiment, blood samples were collected from the caudal vein of five fish of all treatments and were divided into two portions. The first portion was collected with anticoagulant 10% EDTA (ethylenediaminetetraacetate) to measure hematocrit (Ht), hemoglobin (Hb), and white blood cells (WBCs). Ht was determined as described by [42]. Hemoglobin (Hb) was determined by the hemoglobin kits which is a standardized procedure of the cyanomet hemoglobin method. The second portion of the blood sample was allowed to clot overnight at 4ºC and centrifuged at 3,000 rpm for 10 min. The non-hemolyzed serum was collected and stored at −20 ºC until use. Levels of serum aspartate aminotransferase (AST) and alanine aminotransferase (ALT) were estimated according to the method described by [42]. Serum creatinine and uric acid were measured by calorimetric and enzymatic determination methods as described by [43]. Total serum protein and albumin and globulin were determined according to [44].

### Immune responses

The level of serum total immunoglobulin M (IgM) was determined by an ELISA assay kit (Cusabio, Wuhan, Hubei, China). The test kits were purchased from Shenzhen Mindray Bio-medical Electronics Co., Ltd. Lysozyme activity was determined with the turbidimetric method [45] modified by [46]. Non-clotting blood samples were used to estimate leukocyte phagocytic function according to the method of [47].

### Assessments of the liver’s antioxidant activity

Hepatic samples (livers of three fish per replicate) were weighed, homogenized, and rinsed with ice-cold phosphate buffer (1:10; phosphate buffer: pH 7.4, 0.064 M) after anesthetizing the fish with 3-aminobenzoic acid ethyl ester (MS 222, 100 mg/L, Sigma, St. Louis, MO). Based on the [48] method, the homogenate was centrifuged for 10 min at 4°C and 4000 g, and the supernatant was used to assay the activity of superoxide dismutase (SOD). According to [49], the concentration of malondialdehyde (MDA) was assessed. A modified technique of [50] was used to assess the catalase (CAT) activity. The activities of glutathione peroxidase (GPx) and glutathione (GSH) were assessed according to [51] and [52], respectively.

### Gene expression

After fish were anesthetized by using 3-aminobenzoic acid ethyl ester (MS 222, 100 mg/L, Sigma, St. Louis, MO), liver samples from three fish for each treatment were removed from all studied treatments as well as the control and homogenized by Tissue Lyser LT apparatus (QIAGEN; Cat No./ID: 85,600). Total ribonucleic acid (RNA) was extracted from these tissues using RNeasy® Mini kit (Qiagen, Cat No. 74104), based on the manufacturer’s protocol provided in the kit. The reverse transcriptase reaction of RNA was conducted for complementary DNA (cDNA) synthesis according to the protocol of the High-Capacity cDNA Reverse Transcription Kit (Thermo Fisher Scientific, Waltham, MA), cDNA was stored at −80°C for further molecular analyses. Primers of target genes; interleukin 1β (*IL-1β*), interferon-gamma (*IFN-γ*), and heat shock protein 70 (*hsp*70) genes and 18s rRNA as a housekeeping gene were noted in Table 2. The quantitative PCR reaction contained 2.5 μl of 1 μg/μl cDNA, 12.5 μl SimplyGreen SYBR Green qPCR Master Mix, Low Rox (Cat SQ102−0100, GeneDireX, Inc), 0.3 μM of each of forward and reverse primers, 1 μl RNase inhibitor, and RNase-free water to a final volume of 25 μl. The reaction was run on an AriaMax Real-Time PCR (Agilent Technologies, USA) using a two steps protocol: hot-start at 95°C for 10 min, followed by 40 cycles of denaturation at 95°C for 15 s and annealing/extension at 60°C for 1 min, and ending with a melt curve from 65 to 95°C. The expression levels of selected target genes were normalized to those of the *18*_*S*_ *rRNA* gene. Changes in expression levels of the target genes were presented as n-fold changes relative to the corresponding controls. Relative gene expression ratios (RQ) were estimated using the formula: RQ = 2^- ΔΔCT^ [53].

**Table 2:** Oligonucleotide name and sequence of qRT-PCR primers used in this experiment.

Gene	Primer sequence 5′-3′	Amplicon Size (bp)	Slope	Efficiency (%)	R²	Accession No.
18s	F:5’-GGTTGCAAAGCTGAAACTTAAAGG-3’	85 bp	−3.32	100	0.998	AF497908.1
	R:5’TTCCCGTGTTGAGTCAAATTAAGC-3’	256 bp	−3.40	96	0.997	
Interferon-gamma (*IFN-γ*)	F:5’-AAGAATCGCAGCTCTGCACCAT-3’	296 bp	−3.25	103	0.995	XM_005448319.1
	R:5’-GTGTCGTATTGCTGTGGCTTCC-3	107 bp	−3.50	93	0.999	
Interleukin 1β (*IL-1β*)	F:5’-CAAGGATGACGACAAGCCAACC-3′	85 bp	−3.32	100	0.998	XM_003460625.2
	R:5’-AGCGGACAGACATGAGAGTGC-3′	256 bp	−3.40	96	0.997	
heat shock protein 70 (*hsp*70)	F:5’-CATCGCCTACGGTCTGGACAA-3′	296 bp	−3.25	103	0.995	′ FJ207463.1
	R:5’-TGCCGTCTTCAATGGTCAGGAT-3′	107 bp	−3.50	93	0.999	

The primers efficiency was evaluated in UCSC In-Silico PCR on https://genome.ucsc.edu/cgi-bin/hgPcr.

### Statistical analysis of data

Data were tested for homogeneity and normality tests. Afterwards, data were analyzed using one-way analysis of variance and the differences among means were made by using Duncan’s multiple range test using SAS ANOVA procedure [54]. The differences at P < 0.05 were considered significant. The values are presented as means± standard error of the mean (SEM).

## Results

### Growth performance and feed utilization

Dietary fucoidan with different levels significantly (P < 0.05) enhanced the performance of WG, SGR, and FCR of Nile tilapia (Table 3). Fish fed diet supplemented with 1.5 mg kg^-1^ diet fucoidan noted the highest WG, SGR, and PER compared with the control diets. Furthermore, the best FCR was obtained by fish fed 1.5 mg kg^-1^ diet fucoidan, which significantly (P < 0.05) recorded the best values of FCR values in comparison with the control diet, while no significant (P > 0.05) differences were found in FCR between 1 mg and 1.5 mg kg^-1^ diet fucoidan. Fish survival in fish fed diets supplemented with different levels of fucoidan was significantly higher (P < 0.05) than control diet (Table 3), while the best fish survival was recorded in diets 1 and 1.5 mg kg^-1^ diet fucoidan.

**Table 3:** Growth performance and feed utilization of Nile tilapia fed different levels of fucoidan.

Items	Experimental Diets					P-Value
	Control	0.5 mg kg^-1^	1.0 mg kg^-1^	1.5 mg kg^-1^	2.0 mg kg^-1^	
Initial body weight (g fish ^−1^)	2.53 ± 0.22	2.59 ± 0.41	2.52 ± 0.31	2.60 ± 0.23	2.42 ± 0.22	0.069
Final body weight (g fish ^−1^)	21.90 ± 1.2^c^	26.92 ± 1.4^b^	27.92 ± 0.91^b^	29.36 ± 1.2^a^	30.11 ± 1.2^a^	0.025
Weight gain (g fish ^−1^)	19.37 ± 1.2^c^	24.33 ± 1.3^b^	25.43 ± 1.4^b^	26.80 ± 1.5^a^	27.69 ± 1.3^a^	0.002
Specific growth rate (%day ^−1^)	3.08 ± 0.14^c^	3.34 ± 0.41^b^	3.44 ± 0.21^a^	3.49 ± 0.11^a^	3.60 ± 0.13^a^	0.019
Feed conversion ratio	1.69 ± 0.22^a^	1.57 ± 0.14^b^	1.55 ± 0.25^b^	1.47 ± 0.34^c^	1.46 ± 0.25^c^	0.004
Protein efficiency ratio	1.81 ± 0.12^c^	1.94 ± 0.11^b^	1.98 ± 0.13^b^	2.03 ± 0.11^a^	2.06 ± 0.11^a^	0.021
Fish survival (%)	96 ± 1.73^c^	97 ± 1.34^b^	98 ± 1.85^b^	99 ± 1.98^a^	99 ± 1.93^a^	0.012

Means followed by different letters in the same row are significantly different (*P <* 0.05).

### Intestinal tract morphometry

The length of villus, intervilli distance, and the goblet cells number in the middle intestines were significantly (P < 0.05) improved by different dietary levels of fucoidan (Table 4). The highest width and length of villus and inter villi distance were detected in fish fed diet supplemented with 2 mg kg-1 diet fucoidan. The highest number of goblet cells was detected in fish fed diet supplemented with 1.5 mg and 2 mg kg-1 diet fucoidan with no significant differences.

**Table 4:** Intestinal tract morphology of Nile tilapia fed different levels of fucoidan.

Items	Experimental diets					P- value
	Control	0.5 mg kg^-1^	1.0 mg kg^-1^	1.5 mg kg^-1^	2.0 mg kg^-1^	
Villi length (μm)	281.81 ± 5.3^c^	302.10 ± 5.9^c^	351.40 ± 8.3^b^	383.50 ± 7.6^b^	432.12 ± 3.3^a^	0.001
Villi width (μm)	80.22 ± 2.8^e^	108.11 ± 4.3^d^	132.11 ± 3.71^c^	172.01 ± 2.26^b^	197.00 ± 4.23^a^	0.011
Intervilli distance (μm)	62.30 ± 1.2^c^	71.33 ± 1.1^b^	81.12 ± 1.7^b^	91.88 ± 1.8^a^	107 ± 1.72^a^	0.005
Goblet cells/mm^2^	15.35 ± 0.84^d^	20.46 ± 0.51^c^	22.63 ± 0.81^b^	23.24 ± 0.91^a^	28.97 ± 0.43^a^	0.007

Means followed by different letters in the same row are significantly different (*P <* 0.05).

### The endogenous enzymes activities

Digestive intestinal enzyme activities are shown in Table 5. The addition of fucoidan with different levels significantly improved the digestive enzymes. The highest activities of chymotrypsin, trypsin, lipase, amylase and alkaline phosphatase were detected in fish fed diet supplemented with 2 mg kg^-1^ diet fucoidan.

**Table 5:** Intestinal digestive enzyme (U/g tissue) of Nile tilapia fed different levels of fucoidan.

Items	Experimental diets					P- value
	Control	0.5 mg kg^-1^	1.0 mg kg^-1^	1.5 mg kg^-1^	2.0 mg kg^-1^	
Chymotrypsin	4.26 ± 1.02^e^	6.36 ± 1.22^d^	8.45 ± 1.87^c^	8.79 ± 1.32^b^	9.66 ± 1.22^a^	0.021
Trypsin	1.18 ± 0.02^c^	1.41 ± 0.03^c^	1.93 ± 0.02^b^	2.36 ± 0.02^b^	3.87 ± 0.08^a^	0.051
Lipase	907 ± 4.62^e^	992 ± 5.79^d^	1202 ± 12.28^c^	1213 ± 4.21^b^	1340 ± 5.12^a^	0.012
Amylase	714 ± 8.42^e^	896 ± 10.52^d^	918 ± 9.12^c^	993 ± 11.12^b^	1108 ± 13.45^a^	0.021
Alkaline phosphatase	21.58 ± 1.52^e^	25.59 ± 0.02^d^	44.27 ± 0.02^c^	53.13 ± 1.02^b^	73.81 ± 8.36^a^	0.025

Means followed by different letters in the same row are significantly different (*P <* 0.05).

### Hematological parameters

Fucoidan supplementation with different levels had a positive significant effect (P > 0.05) on Hb, Htc, RBCs and WBCs count and their differentiation (Table 6). Diets supplemented with 2 mg kg^-1^ diet fucoidan displayed the highest Hb, Htc, RBCs, and WBCs count. However, neutrophil, lymphocyte, monocyte, and eosinophil were significantly higher in fish fed diet supplemented with 0.5 mg, 1.0 mg, 1.5 mg and 2 mg kg^-1^ diet fucoidan than the control.

**Table 6:** Hematological parameters of Nile tilapia fed different levels of fucoidan.

Items	Experimental Diets					P- Value
	Control	0.5 mg kg^-1^	1.0 mg kg^-1^	1.5 mg kg^-1^	2.0 mg kg^-1^	
Hb (g dl^-1^)	9.66 ± 0.92^d^	11.56 ± 0.82^c^	10.05 ± 0.77^b^	13.89 ± 0.22^a^	14.36 ± 0.32^a^	0.020
Hct (%)	26.21 ± 0.64^c^	29.22 ± 0.54b	29.12 ± 0.61^b^	31.28 ± 0.01^a^	31.78 ± 0.39^a^	0.001
RBCs × 10^6^ mm^3^	2.79 ± 0.32^b^	2.97 ± 1.19^b^	3.32 ± 0.11^a^	3.45 ± 0.23^a^	3.66 ± 0.22^a^	0.011
WBC × 10^4^ mm^3^	31.21 ± 1.25^c^	36.22 ± 1.24^b^	38.12 ± 2.22^b^	43.19 ± 2.55^a^	45.38 ± 1.87^a^	0.015
Neutrophil %	9.11 ± 0.81^c^	13.12 ± 1.22^a^	13.82 ± 1.06^a^	12.98 ± 1.08^a^	13.6 ± 0.09^a^	0.011
Lymphocyte %	72.23 ± 1.26^c^	78.12 ± 3.15^a^	79.23 ± 4.33^a^	82.12 ± 2.56^a^	83.52 ± 1.59^a^	0.020
Monocyte %	3.13 ± 0.12^b^	5.11 ± 0.32^a^	5.72 ± 0.72^a^	6.31 ± 0.12^a^	6.41 ± 55^a^	0.004
Eosinophil %	1.48 ± 1.22^b^	2.25 ± 0.02^a^	3.31 ± 0.22^a^	3.13 ± 1.02^a^	3.65 ± 0.06^a^	0.011

Means followed by different letters in the same row are significantly different (*P <* 0.05).

### Serum biochemical parameters

Dietary supplements of fucoidan with different levels from 0.5 to 2 mg kg^-1^ diet significantly affect the activities of alanine aminotransferase (ALT) and aspartate aminotransferase (AST) among experimental diets (Table 7). Diets supplemented with different levels of fucoidan had lower levels of ALT and AST than the control. However, a significant (P < 0.05) improvement in the serum total protein, albumin, and globulin was shown in response to the supplementation of dietary fucoidan with different levels (Table 7). The highest values of total protein, albumin, and globulin were noted in diet supplemented with 2 mg kg^-1^ diet fucoidan.

**Table 7:** Serum biochemical indices of Nile tilapia fed different levels of fucoidan.

Items	Experimental Diets					P- Value
	Control	0.5 mg kg^-1^	1.0 mg kg^-1^	1.5 mg kg^-1^	2.0 mg kg^-1^	
Alanine aminotransferase (UL^-1^)	27.23 ± 0.70^a^	25.22 ± 0.50 ^b^	25.20 ± 0.27 ^b^	24.35 ± 0.50^c^	25.6 ± 0.30 ^c^	0.060
Aspartate aminotransferase (UL^-1^)	14.12 ± 0.64 ^a^	14.21 ± 0.84 ^b^	12.40 ± 0.71 ^b^	12.18 ± 0.71 ^b^	12.12 ± 0.89 ^b^	0.062
Total protein (g dL^-1^)	2.21 ± 0.37^d^	2.90 ± 0.35^c^	3.40 ± 0.41^b^	4.12 ± 0.32 ^a^	4.20 ± 0.21^a^	0.011
Albumin (g dL^-1^)	1.06 ± 0.01^c^	1.38 ± 0.03^b^	1.57 ± 0.04^b^	1.60 ± 0.07^a^	1.91 ± 0.02^a^	0.012
Globulin (g dL^-1^)	1.15 ± 0.10^c^	1.52 ± 0.02^b^	1.83 ± 0.07^b^	2.52 ± 0.04^a^	2.29 ± 0.09^a^	0.003

Means followed by different letters in the same row are significantly different (*P <* 0.05).

### Immune parameters responses

Table 8 shows that the phagocytic, lysozyme, IgM, and IgG values were significantly improved in fish fed diets supplemented with fucoidan from 0.5 to 2 mg kg^-1^ diet compared with the control diet and the highest values were found in 2 mg kg^-1^ fucoidan diet.

**Table 8:** Immune response of Nile tilapia, *O. niloticus,* fed different levels of fucoidan.

Items	Experimental Diets					P- Value
	Control	0.5 mg kg^-1^	1.0 mg kg^-1^	1.5 mg kg^-1^	2.0 mg kg^-1^	
IgM Immunoglobulin M (mg dL^-1^)	7.53 ± 0.42^c^	8.01 ± 0.02^c^	11.21 ± 0.52^b^	16.23 ± 1.02^b^	17.15 ± 1.26^a^	0.012
IgG Immunoglobulin M (mg dL^-1^)	14.18 ± 0.92c	16.23 ± 0.12c	18.29 ± 0.92b	21.20 ± 1.91^b^	25.19 ± 1.76^a^	0.032
Phagocytes (%)	21.03 ± 0.56^c^	25.13 ± 0.89^a^	37.53 ± 1.67^b^	40.33 ± 1.23^b^	42.31 ± 2.30^b^	0.011
Lysozyme (U ml^-1^)	141.00 ± 1.30^c^	268.33 ± 2.30^a^	278.33 ± 2.10^b^	287.13 ± 1.15^b^	297.13 ± 12.31^b^	0.001

Means followed by different letters in the same row are significantly different (*P <* 0.05).

### Oxidative stress responses

The application of dietary fucoidan with different levels significantly (P < 0.05) improved the oxidative response enzymes (Table 9). A diet supplemented with 2 mg kg^-1^ diet fucoidan recorded the highest levels of SOD, CAT, GSH, and GPx, but the lowest value of MDA was noted in diet supplemented with 2 mg kg^-1^ fucoidan.

**Table 9:** Hepatic oxidative response (U/g protein) of Nile tilapia, *O. niloticus,* fed different levels of fucoidan.

Items	Experimental Diets					P- Value
	Control	0.5 mg kg^-1^	1.0 mg kg^-1^	1.5 mg kg^-1^	2.0 mg kg^-1^	
MDA	31.2 ± 0.42^a^	25.21 ± 0.12^b^	21.21 ± 0.27^c^	19.35 ± 0.12^d^	17.51 ± 0.52^d^	0.011
CAT	51.30 ± 1.14^c^	60.22 ± 2.84b	66.32 ± 3.11^b^	75.36 ± 2.61^b^	75.51 ± 2.39^a^	0.012
GSH	261.31 ± 7.17^e^	278.21 ± 3.25^d^	281.01 ± 8.41^c^	311.22 ± 6.32^b^	351.61 ± 5.21^a^	0.001
GPX	151.31 ± 6.51^e^	161.35 ± 9.13^d^	186.26 ± 8.24^c^	191.31 ± 8.17^b^	198.80 ± 9.12^a^	0.042
SOD	18.58 ± 1.42^e^	29.55 ± 2.01^d^	31.29 ± 5.12^c^	41.23 ± 1.02^b^	56.15 ± 3.16^a^	0.001

Means followed by different letters in the same row are significantly different (*P <* 0.05).

a MDA, *Malondialdehyde.*

b CAT, *Catalase*.

^c^GSH, *Glutathione.*

^d^GPx, *Glutathione peroxidase*.

^e^SOD, *Superoxide dismutase*.

### Gene expression

**Fig 1:**
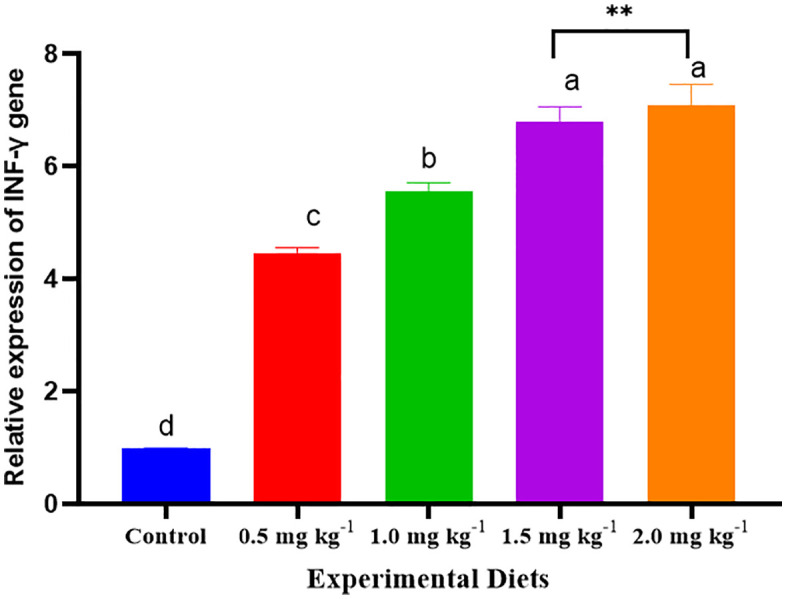
Relative gene expression of INF-γ of Nile tilapia, *O. niloticus* fed diets containing different fucoidan.

**Fig 2:**
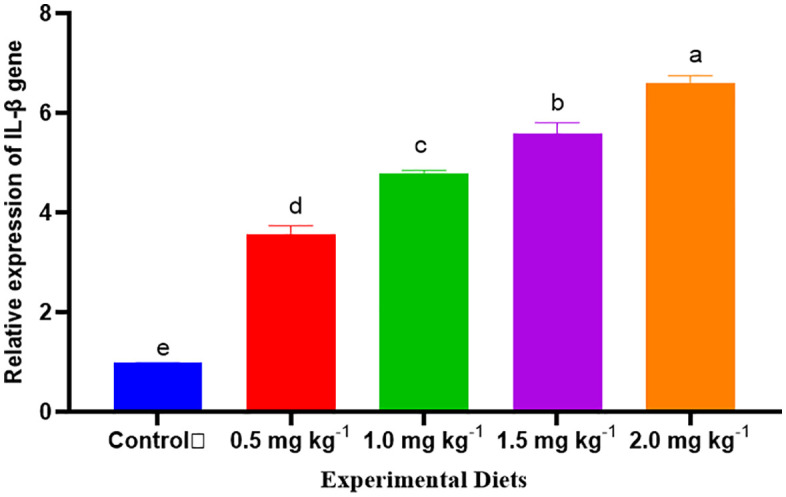
Relative gene expression of IL-1β of Nile tilapia, *O. niloticus* fed diets containing different fucoidan.

**Fig 3:**
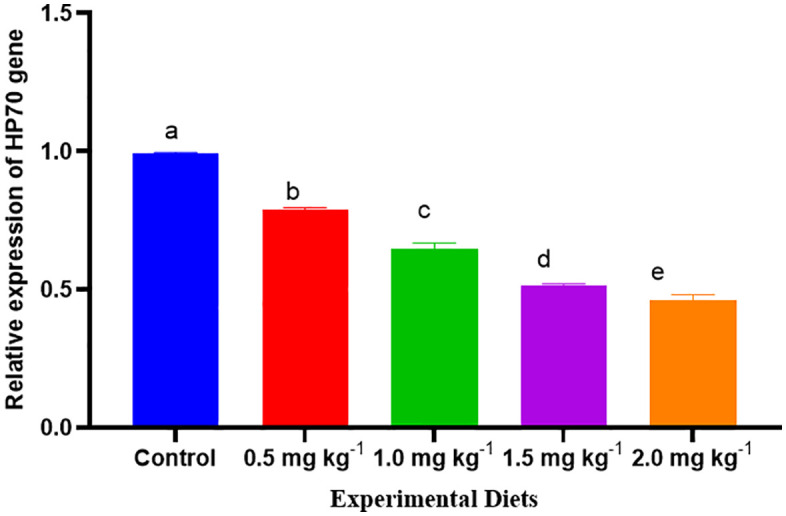
Relative gene expression of HSP70 of Nile tilapia, *O. niloticus* fed diets containing different fucoidan.

Expressions of interferon-gamma (*inf-γ*), interleukin 1β (*il-1β*), and *hp70* genes of fish as affected by fucoidan are presented in Figs 1–3. Fish fed a diet supplemented with different levels of fucoidan significantly (*P* < 0.05) up-regulated interferon-gamma (INF-γ), interleukin 1β (*il-1β*) compared with the control, but the heat shock protein 70 (*hp70*) gene was down-regulated. Diet supplemented with 2 mg kg^-1^ fucoidan displayed the highest gene expression of the *inf-γ* and *il-1β*.

## Discussion

The current study exhibited that dietary inclusion of fucoidan improved the performance, nutrient utilization, and survival rate of Nile tilapia; however, 2 mg kg^-1^ diet fucoidan displayed the highest growth performance of fish. The present results are consistent with [19,55] they found that the inclusion of fucoidan has shown a significant increase in the growth performance of aquatic animals. In line with our results, [56] found an improvement - in growth rate, FCR, and survival rate of Nile tilapia fed diet supplemented with fucoidan. Also, the growth performance and feed utilization parameters of common carp were significantly improved when fish received diet supplemented with fucoidan at a rate of 1,666.67–1,757 mg/kg [57]. Furthermore, fucoidan supplementation significantly enhanced the growth performance of Crayfish [58], red sea bream, *Pagrus major* [59] and black sea bream [60]. Moreover, [61] found that gibel carp, *Carassius gibelio* fed diets supplemented with fucoidan elevated the activity of intestinal digestive enzymes which might consequently improve growth performance and intestine health status. However, [62] found no significant difference in the growth performance of *Labeo rohita* fed diets including different doses of fucoidan. The improvements in growth performance could be attributed to different scenarios such as: i) the stimulatory effect of fucoidan which improves the beneficial intestinal flora’s ability, resulting in improved digestibility, assimilation of nutrients, and digestive enzyme activity [62]., ii) the growth-promoting properties of fucoidan and modulation immune system [57]. The findings stated that fish fed diets supplemented with fucoidan exhibited higher digestive enzyme activity and confirmed by [63]., iii) increased secretions of different digestive enzymes due to sulfated polysaccharides enriched fucoidan inclusion could help to enhance feed utilization efficiency and performance [64].

The present results showed that the highest digestive enzymes’ activity (lipase, trypsin, amylase, and chymotrypsin) of Nile tilapia fed with 2 mg kg^-1^ fucoidan. The present study is consistent with [64] who found that inclusion of fucoidan increased the secretions and activity of different digestive enzymes. Moreover, [61] found that gibel carp fed diets supplemented with fucoidan elevated the activity of intestinal digestive enzymes, which might consequently improve growth performance and intestine health status. Similarly, with our results [57] found that there was higher activity of digestive enzymes in the intestine of common carp when fed diet supplemented with different levels of fucoidan than the control without supplementation. Equally, the present study [61] found that fish received diet inclusion with fucoidan had a higher activity of digestive enzymes. The improvement of digestive enzymes might be due to the up‐regulated expression of Muscarinic acetylcholine receptors (mAChRs) M3 which have vital functions in stimulating digestive enzyme secretion and activity of pancreatic acinar cells [63,65].

Other previous studies found a positive relation between intestinal digestion and intestinal microbial composition [66,67]. This may be another reason for the improvement of digestive enzymes. In this context, [61] found that fucoidan supplementation significantly improved the intestinal microbiota composition of gibel carp. Also, fucoidan significantly increased the abundance of Aeromonas from approximately 8% to 13% that consequently elevated the activity of digestive enzymes [61].

Fish fed fucoidan, either 1.5 or 2 g kg^-1^ had significantly longer and wider intestinal villi and a higher number of goblet cells than the untreated group. These outcomes are consistent with [61] who found that gibel carp-fed diet containing 30 g/kg fucoidan had a significantly higher abundance of goblet cells. In line with the present study, [68] found that Nile tilapia fed diet supplemented with fucoidan had higher intestinal length, width, villi surface area, and number of intra-epithelial lymphocytes. Similarly, [69,70] found that supplementation of fucoidan improves gut health of pigs. Moreover, [71] revealed that common carp (*Cyprinus carpio*) fed a diet containing β-glucan polysaccharide increased the mucin-containing goblet cells.

Hematological parameters such as red blood cell count (RBCs), hemoglobin (Hb), and hematocrit (Hct) are fundamental indicators of fish health, oxygen transport capacity, and physiological status [41,72]. The levels of Hb and RBCs indicate the anemic and respiration capacity of blood cells, whereas hematocrit and WBCs levels display the immunological status of fish [73]. The present study indicated that diets supplemented with 2 mg kg^-1^ diet fucoidan displayed the highest values of Hb, Htc, RBCs, and WBCs. Consistent with the present study, [56] found that the highest hematological indices such as Hb, PCV, RBCs, and WBCs, were observed in Nile tilapia fed diet inclusion with fucoidan. Moreover, the hematocrit of red sea bream (*Pagrus major*) increased numerically with the supplementation of fucoidan [59].

Biochemical blood factors are diagnostic tools for evaluating the nutritional status, health status, and immune response of fish [72,74]. Plasma AST and ALT are indicators of liver function as they are released into the blood during injury or damage to the liver cells [75]. In the present study, fish fed diets supplemented with either 1.5 or 2 mg kg^-1^ fucoidan displayed the best values of liver enzymes ALT and AST compared to the control. These findings herein reflect the positive effect of fucoidan supplementation on the liver health of Nile tilapia. In line with our results, [76] found that fucoidan supplementation improved the liver enzymes in Nile tilapia. Also, the present study exhibited higher values of total protein, albumin, and globulin in tilapia fed a diet supplemented with fucoidan compared to the control. In this sense, Fucoidan supplements are recognized for their functionality as metabolic and immunological mediators involved in enhancing the health status and welfare of aquatic animals [77]. Furthermore, fucoidan adjusts the metabolic function and level of proteins and nutrients in the blood, leading to high proteins and immune-related factors [78]. Synchronized with the present study, dietary fucoidan regulated the metabolites in Nile tilapia [76]. Also, the present results are in parallel with [59] who concluded that the optimal levels of dietary fucoidan supplementation around 0.3 and 0.4% for juvenile red sea bream improved the activities of ALT and AST as well as serum total protein, which was significantly improved in fish fed diet supplemented with different levels of fucoidan.

The antioxidant system is the first line of defense for fish against oxidative stress [23,79,80]. Fucoidan dietary supplements dramatically increased the activity of CAT, GPx, and SOD in our study versus the basal diet, indicating their function as antioxidants. Similar results have also been reported in *Cyprinus carpio* [59] who stated that inclusion of fucoidan enhanced oxidative enzymes activity and oxidative stress resistance. Consistent with our results, the antioxidant enzymes including CAT, GPx, and, MDA were significantly improved in Nile tilapia fed diet supplemented with fucoidan (Abdel-Warith et al, 2021). Also, [57] reported that diet inclusion with fucoidan increased the activities of SOD, CAT, POD, and GPX, while decreasing the activities of MDA in common carp. In the same sense fucoidan supplementation improved the activities of antioxidant enzymes in different aquatic animals, including yellow catfish [20], whiteleg shrimp [81]. Moreover, [82] reported that inclusion of fucoidan decrease the serum MDA contents in mice. In addition, [20] also found significantly lower MDA content in fed diets supplemented with fucoidan. This suggests that fucoidan plays an important role in enhancing the activity of antioxidant enzymes.

Lysozyme, IgM, IgG, and phagocytes are good indictors of the nonspecific and specific immunity of fish and also improved natural protective mechanisms in fish [83,84]. In the present study, fish fed diets supplemented with 2 mg kg^-1^ diet fucoidan recorded the highest values of IgM and IgG, while fish fed 0.5 g kg^-1^ diet fucoidan recorded the highest phagocytes and lysozyme. Similarly, with our results, fucoidan supplementation significantly improved the activity of lysozyme, IgM of common carp. Compared to control without supplementation [57]. In the same context, [85] found higher activities of immune responses such as lymphocytes and granulocyte numbers, phagocytic, and lysozyme in common carp treated with fucoidan. Also, the same effect of fucoidan on immune response parameters was recorded in Japanese flounder (*Paralichthys olivaceus*) [86], and yellow catfish (*Pelteobagrus fulvidraco*) [20]. The improvement of immune response parameters in the present study may be due to the antioxidant, anti-inflammatory, and immunomodulatory effects of fucoidan [87–90].

Interferon-gamma (*inf-γ*) and interleukin 1β (*il-1β*) are good indicators for the immune response adjustment [87,88], while *hp70* genes are important as inflammation markers and stress [91,92]. In the present study, fucoidan supplementation significantly (P < 0.05) improved the expressions of interferon-gamma (*inf-γ*), interleukin 1β (*il-1β*), and *hp70* genes. Present study displayed up-regulated interferon-gamma (*inf-γ*) and interleukin 1β (*il-1β*) in fish that received a diet with fucoidan compared to the control which have important roles in the innate immune system. Consistent with our results, [55] found that fucoidan supplementation from *S. wightii* increased the expression of interferon-gamma (*INF-γ*) in striped catfish fingerlings. Moreover, the addition of an appropriate level of fucoidan in the common carp diet up-regulates the expression of *IL-6* and *IL-1b* and *IL-10* genes, which are related to inflammation and a proinflammatory effect [57]. Also, [61] found a positive effect of fucoidan supplementation on the expression of genes involved in immune regulation (such as interleukin‐8 and cyclooxygenase) of gibel carp. Fucoidan supplementation might stimulate the expression of proinflammatory mediators and lead to improvement of immune readiness of the host [61].

### Conclusion

According to the results, supplementing the diet with fucoidan increased digestive enzyme activity, feed utilization, growth performance, intestinal histology, hematological parameters, serum biochemical parameters, and antioxidant enzyme activities in Nile tilapia (*Oreochromis niloticus*). This suggests that fish-fed fucoidan at different levels was healthier than the control group. Nevertheless, deeper research is essential to explore the effects of fucoidan on diverse fish species.
