# Supplementary material for: Can dietary supplementation with highly purified fucoidan alter growth, digestive enzyme activity, serum biochemicals, immune-antioxidant responses, and related gene expressions in Nile tilapia (Oreochromis niloticus)?
Source: PLoS One. 2026 Jul 10;21(7):e0339270. doi: 10.1371/journal.pone.0339270 (PMC13354009; doi:10.1371/journal.pone.0339270)
Supplement: Peer review history

# Peer review: Decision Letter 0

**DOI:** 10.1371/journal.pone.0339270.r001

**Authors:** Lee Seong

12 Oct 2025

PONE-D-25-52195

Can dietary supplementation a highly purified fucoidan alter growth, digestive enzyme activity, serum biochemicals, and immune-antioxidant response and related gene expressions of Nile tilapia?

PLOS ONE

Dear Dr. Hassaan,

Thank you for submitting your manuscript to PLOS ONE. After careful consideration, we feel that it has merit but does not fully meet PLOS ONE’s publication criteria as it currently stands. Therefore, we invite you to submit a revised version of the manuscript that addresses the points raised during the review process.

Please submit your revised manuscript by Nov 26 2025 11:59PM. If you will need more time than this to complete your revisions, please reply to this message or contact the journal office at plosone@plos.org. When you’re ready to submit your revision, log on to https://www.editorialmanager.com/pone/ and select the 'Submissions Needing Revision' folder to locate your manuscript file.

Please include the following items when submitting your revised manuscript:

- A rebuttal letter that responds to each point raised by the academic editor and reviewer(s). You should upload this letter as a separate file labeled 'Response to Reviewers'.
- A marked-up copy of your manuscript that highlights changes made to the original version. You should upload this as a separate file labeled 'Revised Manuscript with Track Changes'.
- An unmarked version of your revised paper without tracked changes. You should upload this as a separate file labeled 'Manuscript'.

If you would like to make changes to your financial disclosure, please include your updated statement in your cover letter. Guidelines for resubmitting your figure files are available below the reviewer comments at the end of this letter.

If applicable, we recommend that you deposit your laboratory protocols in protocols.io to enhance the reproducibility of your results. Protocols.io assigns your protocol its own identifier (DOI) so that it can be cited independently in the future. For instructions see: https://journals.plos.org/plosone/s/submission-guidelines#loc-laboratory-protocols. Additionally, PLOS ONE offers an option for publishing peer-reviewed Lab Protocol articles, which describe protocols hosted on protocols.io. Read more information on sharing protocols at https://plos.org/protocols?utm_medium=editorial-email&utm_source=authorletters&utm_campaign=protocols.

We look forward to receiving your revised manuscript.

Kind regards,

Lee Seong

Academic Editor

PLOS ONE

Journal Requirements:

When submitting your revision, we need you to address these additional requirements.

1. Please ensure that your manuscript meets PLOS ONE's style requirements, including those for file naming. The PLOS ONE style templates can be found at https://journals.plos.org/plosone/s/file?id=wjVg/PLOSOne_formatting_sample_main_body.pdf and https://journals.plos.org/plosone/s/file?id=ba62/PLOSOne_formatting_sample_title_authors_affiliations.pdf

2. To comply with PLOS One submissions requirements, in your Methods section, please provide additional information regarding the experiments involving animals and ensure you have included details on (1) methods of sacrifice, (2) methods of anesthesia and/or analgesia, and (3) efforts to alleviate suffering.

3. We note that your Data Availability Statement is currently as follows: All relevant data are within the manuscript and its Supporting Information files

Please confirm at this time whether or not your submission contains all raw data required to replicate the results of your study. Authors must share the “minimal data set” for their submission. PLOS defines the minimal data set to consist of the data required to replicate all study findings reported in the article, as well as related metadata and methods (https://journals.plos.org/plosone/s/data-availability#loc-minimal-data-set-definition).

For example, authors should submit the following data:

- The values behind the means, standard deviations and other measures reported;

- The values used to build graphs;

- The points extracted from images for analysis.

Authors do not need to submit their entire data set if only a portion of the data was used in the reported study.

If your submission does not contain these data, please either upload them as Supporting Information files or deposit them to a stable, public repository and provide us with the relevant URLs, DOIs, or accession numbers. For a list of recommended repositories, please see https://journals.plos.org/plosone/s/recommended-repositories.

If there are ethical or legal restrictions on sharing a de-identified data set, please explain them in detail (e.g., data contain potentially sensitive information, data are owned by a third-party organization, etc.) and who has imposed them (e.g., an ethics committee). Please also provide contact information for a data access committee, ethics committee, or other institutional body to which data requests may be sent. If data are owned by a third party, please indicate how others may request data access.

4. Please amend your authorship list in your manuscript file to include author Mohamed Hassaan, A. Elashry.

5. Please amend the manuscript submission data (via Edit Submission) to include author Mohamed S. Hassaan, Mohamed, A. Elashry.

6. We note you have included a table to which you do not refer in the text of your manuscript. Please ensure that you refer to Table 2 in your text; if accepted, production will need this reference to link the reader to the Table.

7. If the reviewer comments include a recommendation to cite specific previously published works, please review and evaluate these publications to determine whether they are relevant and should be cited. There is no requirement to cite these works unless the editor has indicated otherwise.

[Note: HTML markup is below. Please do not edit.]

Reviewers' comments:

Reviewer's Responses to Questions

**Comments to the Author**

1. Is the manuscript technically sound, and do the data support the conclusions?

The manuscript must describe a technically sound piece of scientific research with data that supports the conclusions. Experiments must have been conducted rigorously, with appropriate controls, replication, and sample sizes. The conclusions must be drawn appropriately based on the data presented.

Reviewer #1: Yes

Reviewer #2: Yes

Reviewer #3: Yes

**********

2. Has the statistical analysis been performed appropriately and rigorously?

Reviewer #1: Yes

Reviewer #2: Yes

Reviewer #3: Yes

**********

3. Have the authors made all data underlying the findings in their manuscript fully available?

The PLOS Data policy requires authors to make all data underlying the findings described in their manuscript fully available without restriction, with rare exception (please refer to the Data Availability Statement in the manuscript PDF file). The data should be provided as part of the manuscript or its supporting information, or deposited to a public repository. For example, in addition to summary statistics, the data points behind means, medians and variance measures should be available. If there are restrictions on publicly sharing data—e.g. participant privacy or use of data from a third party—those must be specified.

Reviewer #1: Yes

Reviewer #2: Yes

Reviewer #3: Yes

**********

4. Is the manuscript presented in an intelligible fashion and written in standard English?

PLOS ONE does not copyedit accepted manuscripts, so the language in submitted articles must be clear, correct, and unambiguous. Any typographical or grammatical errors should be corrected at revision, so please note any specific errors here.

Reviewer #1: Yes

Reviewer #2: No

Reviewer #3: Yes

**********

5. Review Comments to the Author

Please use the space provided to explain your answers to the questions above. You may also include additional comments for the author, including concerns about dual publication, research ethics, or publication ethics. (Please upload your review as an attachment if it exceeds 20,000 characters)

Reviewer #1: This manuscript investigates fucoidan supplementation effects on Nile tilapia growth, physiology, and immune responses. While the topic is relevant to aquaculture, the manuscript requires substantial revisions before publication consideration.

My concerns:

1. Sample Size Ambiguity:

-Lines 100-101 state fish were "divided into three groups with three replicates" but Table 3 and throughout show FIVE treatment groups (Control + 4 fucoidan levels)

-This fundamental inconsistency raises serious questions about the actual experimental design

QUESTION: Was this a 3-group or 5-group study? How many total tanks were used?

Stocking Density Concerns:

-20 fish in 150L tanks = 7.5L per fish initially, but final weights of 30g would make this extremely crowded

-No water quality data provided despite claim parameters were "maintained within recommended range"

-Could Provide actual water quality measurements (DO, ammonia, nitrite, pH, temperature) if possible

2. Methodological Gaps

Fucoidan Characterization Missing:

No molecular weight, purity, or sulfate content data for the fucoidan used

These properties critically affect bioactivity

Dose Selection Not Justified:

Why were 0.5, 1.0, 1.5, 2.0 mg/kg chosen?

Previous studies cited used 1% (10,000 mg/kg) - your doses are 5,000-10,000× lower

QUESTION: Are these doses realistic? Is there a unit error (should it be g/kg not mg/kg)?

Sampling Protocols Unclear:

"Three fish from each tank" for histology but "four fish" for enzyme analysis (line 120)

Were these same or different fish?

24-hour fasting before sampling not mentioned

3. Results Interpretation Problems

Biological Significance vs. Statistical Significance:

Many "significant" differences are trivially small

Example: WBC count 32.21 vs 45.38 (×10⁴/mm³) - is this biologically meaningful?

Growth differences of 1-2g between adjacent treatments may not be economically significant

Inconsistent Dose-Response:

Some parameters show linear increase (growth), others plateau (survival)

No discussion of optimal dose determination

Claims of "best" performance at 2.0 mg/kg lack statistical support (often not different from 1.5 mg/kg)

QUESTIONS:

-Feed Manufacturing: How was fucoidan mixed into feeds? Was stability verified? Storage conditions?

-Intestinal Morphometry (Table 4): How many villi measured per fish? Were measurements blinded?

-Enzyme Assays (Table 5): Were samples run in duplicate/triplicate? What were intra-assay CVs?

-Hematology (Table 6): Manual or automated counting? Differential count method?

-Gene Expression: Why were these three genes selected? What about IL-10, TNF-α, other immunity genes?

-Challenge Test: Why was no pathogen challenge performed to validate immunostimulant claims?

Some minor comments

Abstract & Introduction

Line 28: "Fish weighing 2.51±0.12" - missing units

Line 51: "water pollution with a negative impact of human activities" - awkward phrasing

Line 68: "Fucoidan is a natural polysaccharide" - needs "found in" or "extracted from"

Materials & Methods

Line 108: "A basal diet (310 g kg⁻¹ crude protein)" - inconsistent with Table 1 showing 316.35g/kg

Discussion

Line 254: "however, 2 mg kg⁻¹ diet fucoidan displayed the highest" - sentence fragment

Line 274: "Ozório et al. 2015" - inconsistent citation format (should be in parentheses)

Line 349: "IgM" appears without definition earlier in text

Some literatures should be used to enhance this manuscript:

-https://www.sciencedirect.com/science/article/abs/pii/S0377840125001130

-https://link.springer.com/article/10.1007/s10499-025-01960-7

-https://link.springer.com/article/10.1007/s10499-025-02205-3

Reviewer #2: The study is well written with obvious results..

The following adjustments are suggested during the revision:

-Abstract section have to cover your goal of this manuscript rewrite this section

-Abstract section is too long also compare treatments with p value in this section

-In introduction section, add more information about role immunostimulatns as feed addative on aquatics animals pleaseconsider below publications:

Benefits of Dietary Polyphenols and Polyphenol-Rich Additives to Aquatic Animal Health: An Overview

Dietary plant pigment on blood-digestive physiology, antioxidant-immune response, and inflammatory gene transcriptional regulation in spotted snakehead (Channa punctata) infected with Pseudomonas aeruginosa

Can dietary ginger (Zingiber officinale) alter biochemical and immunological parameters and gene expression related to growth, immunity and antioxidant system in zebrafish (Danio rerio)?

-Extract of grape seed enhances the growth performance, humoral and mucosal immunity, and resistance of common carp (Cyprinus carpio) against Aeromonas hydrophila

Cornelian cherry (Cornus mas L.) fruit extract improves growth performance, disease resistance, and serum immune-and antioxidant-related gene expression of common carp (Cyprinus carpio)

Effects of dietary Plantago ovata seed extract administration on growth performance and immune function of common carp (Cyprinus carpio) fingerling exposed to ammonia toxicity

-In line 109, according to which refrences select these dose for incorporation?

-In table 2, write name of gene as completely

-This manuscript need to be revise as English grammer

-

Reviewer #3: Plos One

Manuscript Title: Can dietary supplementation a highly purified fucoidan alter growth, digestive enzyme activity, serum biochemicals, and immune-antioxidant response and related gene expressions of Nile tilapia?

Manuscript Number: PONE-D-25-52195

General comment: Empirical data strongly supports the manuscript, making a unique contribution. Before publication, minor changes are necessary that should be made are shown below.

1)Please revise the title to be “Can dietary supplementation with highly purified fucoidan alter growth, digestive enzyme activity, serum biochemicals, immune-antioxidant responses, and related gene expressions in Nile tilapia (Oreochromis niloticus)?”

2)Line 28: Add the replicate number and the total number of fish used in the experiment.

3)Line 35: Replace “versus to basal diet” with “compared to the basal diet.”

4)Line 43: Please use keywords different from the title. For instance, you may use: immune response, gene expression, growth performance, antioxidant enzymes, dietary supplementation.

5)The first paragraph of the introduction contains several grammatical and structural issues. Please revise. For example Lines 50-51: “Shifting from extensive to intensive aquaculture systems increases vulnerability to pathogen infections, water pollution, and stress.”

6)The introduction heavily relies on outdated literature. Most references are over a decade old. The manuscript would benefit from a comprehensive update using recent, relevant research from the past five years as possible.

7)Please italicize all scientific names of species (e.g., P. monodon).

8)Line 97: Please specify the scientific name of Nile tilapia (Oreochromis niloticus) the first time it appears in this section.

9)Clarify the source of the fish (e.g., local hatchery, supplier name, or region).

10)Line 101: The Abstract mentions five groups, while this section states “three groups.” Please verify and correct.

11)Line 106: Add water quality parameters (temperature, pH, dissolved oxygen, ammonia, etc.) and the range maintained during the experiment.

12)Table 1: The calculation of Nitrogen-Free Extract is incorrect; please revise it and then adjust the Gross Energy accordingly.

13)Throughout the manuscript, fish gene names should be written in lowercase italics (e.g., il-1β, ifn-γ, hsp70), while protein names should appear in uppercase, non-italicized font (e.g., IL-1β, IFN-γ, HSP70). Please revise the manuscript accordingly to maintain proper scientific formatting and consistency.

14)Table 5: The significance letters indicating statistical differences should be written as superscripts. Please revise the table accordingly to ensure proper formatting.

**********

6. PLOS authors have the option to publish the peer review history of their article (what does this mean?). If published, this will include your full peer review and any attached files.

If you choose “no”, your identity will remain anonymous but your review may still be made public.

**Do you want your identity to be public for this peer review?**  For information about this choice, including consent withdrawal, please see our Privacy Policy.

Reviewer #1: No

Reviewer #2: No

Reviewer #3: No

**********

[NOTE: If reviewer comments were submitted as an attachment file, they will be attached to this email and accessible via the submission site. Please log into your account, locate the manuscript record, and check for the action link "View Attachments". If this link does not appear, there are no attachment files.]

While revising your submission, please upload your figure files to the Preflight Analysis and Conversion Engine (PACE) digital diagnostic tool, https://pacev2.apexcovantage.com/. PACE helps ensure that figures meet PLOS requirements. To use PACE, you must first register as a user. Registration is free. Then, login and navigate to the UPLOAD tab, where you will find detailed instructions on how to use the tool. If you encounter any issues or have any questions when using PACE, please email PLOS at figures@plos.org. Please note that Supporting Information files do not need this step.

# Author comment: Author response to Decision Letter 1

**DOI:** 10.1371/journal.pone.0339270.r002

14 Nov 2025

Response Letter

Reviewer #1:

This manuscript investigates fucoidan supplementation effects on Nile tilapia growth, physiology, and immune responses. While the topic is relevant to aquaculture, the manuscript requires substantial revisions before publication consideration.

Comment 1# -Lines 100-101 state fish were "divided into three groups with three replicates" but Table 3 and throughout show FIVE treatment groups (Control + 4 fucoidan levels)

-This fundamental inconsistency raises serious questions about the actual experimental design QUESTION: Was this a 3-group or 5-group study? How many total tanks were used?

Answer: authors corrected it 5-group represented 15-auarium were used

Comment 2# 20 fish in 150L tanks = 7.5L per fish initially, but final weights of 30g would make this extremely crowded

-No water quality data provided despite claim parameters were "maintained within recommended range"

-Could Provide actual water quality measurements (DO, ammonia, nitrite, pH, temperature) if possible

Answer:

Answer: authors changed 20% of water from each aquarium daily, also added the water quality measurements :During the 70 days feeding trail, the water quality parameters averaged (±SD): water temperature 27.2±0.8 °C: dissolved oxygen, 5.7±0.3 mg L-1: total ammonia, 0.20±0.11 mg L-1 nitrite, 0.07± 0.03 mg L-1: total alkalinity, 169±42 mg L-1: chlorides, 565±152 mg L-1: pH 8.6±0.3.Water quality parameters were maintained within the recommended range of Nile Tilapia according to (Boyd, 1990).

Comment 3# Fucoidan Characterization Missing: No molecular weight, purity, or sulfate content data for the fucoidan used. These properties critically affect bioactivity Dose Selection Not Justified: Why were 0.5, 1.0, 1.5, 2.0 mg/kg chosen? Previous studies cited used 1% (10,000 mg/kg) - your doses are 5,000-10,000× lower QUESTION: Are these doses realistic? Is there a unit error (should it be g/kg not mg/kg)?

Answer: authors added the purity of fucoidan and how added to pellets

Comment 4# "Three fish from each tank" for histology but "four fish" for enzyme analysis (line 120) Were these same or different fish? 24-hour fasting before sampling not mentioned

Answer: authors used different fish for histology and enzymes analysis, as well as fish were fasted 24-hour before sampling.

Comment 5# Biological Significance vs. Statistical Significance: Many "significant" differences are trivially small Example: WBC count 32.21 vs 45.38 (×10⁴/mm³) - is this biologically meaningful? Growth differences of 1-2g between adjacent treatments may not be economically significant.

Answer: authors used data were analyzed using one-way analysis of variance and the differences among means were made by using Duncan's multiple range test using SAS ANOVA procedure (SAS, version 6.03, Soft Inc., Tusla, OK, USA, SAS, 1993).

Comment 6# Some parameters show linear increase (growth), others plateau (survival) No discussion of optimal dose determination Claims of "best" performance at 2.0 mg/kg lack statistical support (often not different from 1.5 mg/kg).

Answer: authors noted that the best results were 1.5-2.0 mg kg.

Comment 7# Feed Manufacturing: How was fucoidan mixed into feeds? Was stability verified? Storage conditions?

Answer: authors added that

Comment 8# Intestinal Morphometry (Table 4): How many villi measured per fish? Were measurements blinded?

Answer: authors noted that in the manuscript

Comment 9# Enzyme Assays (Table 5): Were samples run in duplicate/triplicate? What were intra-assay CVs?

Answer: The samples were triplicate.

Comment 10# Hematology (Table 6): Manual or automated counting? Differential count method?

Answer: authors added the details of blood samples

Comment 11# Gene Expression: Why were these three genes selected? What about IL-10, TNF-α, other immunity genes?

Answer: authors tasted the candidate gene for immunity

Comment 12# Challenge Test: Why was no pathogen challenge performed to validate immunostimulant claims.

Answer: authors thanks reviewer for this comment, but we can do it in future study.

Comment 13# Line 28: "Fish weighing 2.51±0.12" - missing units

Answer: authors added that

Comment 14# Line 51: "water pollution with a negative impact of human activities" - awkward phrasing

Answer: authors paraphrase it

Comment 15# Line 68: "Fucoidan is a natural polysaccharide" - needs "found in" or "extracted from"

Answer: authors corrected it

Comment 16# Line 108: "A basal diet (310 g kg⁻¹ crude protein)" - inconsistent with Table 1 showing 316.35g/kg

Answer: authors corrected it

Comment 17# Line 254: "however, 2 mg kg⁻¹ diet fucoidan displayed the highest" - sentence fragment

Answer: authors completed the sentence

Comment 18# Line 274: "Ozório et al. 2015" - inconsistent citation format (should be in parentheses)

Answer: authors corrected it

Comment 19# Line 349: "IgM" appears without definition earlier in text

Answer: authors corrected it

Reviewer 2

Comment 1# Abstract section is too long also compare treatments with p value in this section

Answer: authors correct it

Comment 2# In introduction section, add more information about role immunostimulatns as feed addative on aquatics animals pleaseconsider below publications:

Benefits of Dietary Polyphenols and Polyphenol-Rich Additives to Aquatic Animal Health: An Overview

Dietary plant pigment on blood-digestive physiology, antioxidant-immune response, and inflammatory gene transcriptional regulation in spotted snakehead (Channa punctata) infected with Pseudomonas aeruginosa

Can dietary ginger (Zingiber officinale) alter biochemical and immunological parameters and gene expression related to growth, immunity and antioxidant system in zebrafish (Danio rerio)?

-Extract of grape seed enhances the growth performance, humoral and mucosal immunity, and resistance of common carp (Cyprinus carpio) against Aeromonas hydrophila

Cornelian cherry (Cornus mas L.) fruit extract improves growth performance, disease resistance, and serum immune-and antioxidant-related gene expression of common carp (Cyprinus carpio)

Effects of dietary Plantago ovata seed extract administration on growth performance and immune function of common carp (Cyprinus carpio) fingerling exposed to ammonia toxicity

Answer: authors used it in introduction

Comment 3# In line 109, according to which refrences select these dose for incorporation?

According to

Comment 4# In table 2, write name of gene as completely

Answer: authors added it

Comment 5# This manuscript need to be revise as English grammar

Answer: authors revised it again

Reviewer 3

Comment 1# Please revise the title to be “Can dietary supplementation with highly purified fucoidan alter growth, digestive enzyme activity, serum biochemicals, immune-antioxidant responses, and related gene expressions in Nile tilapia (Oreochromis niloticus)?”

Answer: authors changed it

Comment 2# Line 28: Add the replicate number and the total number of fish used in the experiment.

Answer: authors added that

Comment 3# Line 35: Replace “versus to basal diet” with “compared to the basal diet.”

Answer: Authors correct it

Comment 4# Line 43: Please use keywords different from the title. For instance, you may use: immune response, gene expression, growth performance, antioxidant enzymes, dietary supplementation.

Answer: authors used it

Comment 5# The first paragraph of the introduction contains several grammatical and structural issues. Please revise. For example Lines 50-51: “Shifting from extensive to intensive aquaculture systems increases vulnerability to pathogen infections, water pollution, and stress.”

Answer: authors changed it

Comment 6# The introduction heavily relies on outdated literature. Most references are over a decade old. The manuscript would benefit from a comprehensive update using recent, relevant research from the past five years as possible.

Answer: authors used recent citation

Comment 7# Please italicize all scientific names of species (e.g., P. monodon).

Answer: authors correct it

Comment 8# Line 97: Please specify the scientific name of Nile tilapia (Oreochromis niloticus) the first time it appears in this section.

Answer: authors correct it

Comment 9# Clarify the source of the fish (e.g., local hatchery, supplier name, or region).

Answer: auteurs added it Nile tilapia O. niloticus were obtained from private farm (Kafer-Elshekh Governorate, Egypt)

Comment 10# Line 101: The Abstract mentions five groups, while this section states “three groups.” Please verify and correct.

Answer: authors corrected it

Comment 11# Line 106: Add water quality parameters (temperature, pH, dissolved oxygen, ammonia, etc.) and the range maintained during the experiment.

Answer: Authors added it

Comment 12# Table 1: The calculation of Nitrogen-Free Extract is incorrect; please revise it and then adjust the Gross Energy accordingly.

Answer: authors thanks reviewer for this comment and we corrected it

Comment 13# Throughout the manuscript, fish gene names should be written in lowercase italics (e.g., il-1β, ifn-γ, hsp70), while protein names should appear in uppercase, non-italicized font (e.g., IL-1β, IFN-γ, HSP70). Please revise the manuscript accordingly to maintain proper scientific formatting and consistency.

Answer: authors corrected it

Comment 14# Table 5: The significance letters indicating statistical differences should be written as superscripts. Please revise the table accordingly to ensure proper formatting.

Answer: authors corrected it

# Peer review: Decision Letter 1

**DOI:** 10.1371/journal.pone.0339270.r003

**Authors:** Lee Seong, Lee Seong

4 Dec 2025

Can dietary supplementation with highly purified fucoidan alter growth, digestive enzyme activity, serum biochemicals, immune-antioxidant responses, and related gene expressions in Nile tilapia (Oreochromis niloticus)?

PONE-D-25-52195R1

Dear Dr. Hassaan,

We’re pleased to inform you that your manuscript has been judged scientifically suitable for publication and will be formally accepted for publication once it meets all outstanding technical requirements.

Within one week, you’ll receive an e-mail detailing the required amendments. When these have been addressed, you’ll receive a formal acceptance letter and your manuscript will be scheduled for publication.

An invoice will be generated when your article is formally accepted. Please note, if your institution has a publishing partnership with PLOS and your article meets the relevant criteria, all or part of your publication costs will be covered. Please make sure your user information is up-to-date by logging into Editorial Manager at Editorial Manager® and clicking the ‘Update My Information' link at the top of the page. For questions related to billing, please contact billing support.

If your institution or institutions have a press office, please notify them about your upcoming paper to help maximize its impact. If they’ll be preparing press materials, please inform our press team as soon as possible -- no later than 48 hours after receiving the formal acceptance. Your manuscript will remain under strict press embargo until 2 pm Eastern Time on the date of publication. For more information, please contact onepress@plos.org.

Kind regards,

Lee Seong

Academic Editor

PLOS One

Additional Editor Comments (optional):

Reviewers' comments:

Reviewer's Responses to Questions

**Comments to the Author**

1. If the authors have adequately addressed your comments raised in a previous round of review and you feel that this manuscript is now acceptable for publication, you may indicate that here to bypass the “Comments to the Author” section, enter your conflict of interest statement in the “Confidential to Editor” section, and submit your "Accept" recommendation.

Reviewer #1: All comments have been addressed

Reviewer #2: All comments have been addressed

Reviewer #3: All comments have been addressed

**********

2. Is the manuscript technically sound, and do the data support the conclusions?

The manuscript must describe a technically sound piece of scientific research with data that supports the conclusions. Experiments must have been conducted rigorously, with appropriate controls, replication, and sample sizes. The conclusions must be drawn appropriately based on the data presented.

Reviewer #1: Yes

Reviewer #2: Yes

Reviewer #3: Yes

**********

3. Has the statistical analysis been performed appropriately and rigorously?

Reviewer #1: Yes

Reviewer #2: Yes

Reviewer #3: Yes

**********

4. Have the authors made all data underlying the findings in their manuscript fully available?

The PLOS Data policy requires authors to make all data underlying the findings described in their manuscript fully available without restriction, with rare exception (please refer to the Data Availability Statement in the manuscript PDF file). The data should be provided as part of the manuscript or its supporting information, or deposited to a public repository. For example, in addition to summary statistics, the data points behind means, medians and variance measures should be available. If there are restrictions on publicly sharing data—e.g. participant privacy or use of data from a third party—those must be specified.

Reviewer #1: Yes

Reviewer #2: Yes

Reviewer #3: Yes

**********

5. Is the manuscript presented in an intelligible fashion and written in standard English?

PLOS ONE does not copyedit accepted manuscripts, so the language in submitted articles must be clear, correct, and unambiguous. Any typographical or grammatical errors should be corrected at revision, so please note any specific errors here.

Reviewer #1: Yes

Reviewer #2: Yes

Reviewer #3: Yes

**********

6. Review Comments to the Author

Please use the space provided to explain your answers to the questions above. You may also include additional comments for the author, including concerns about dual publication, research ethics, or publication ethics. (Please upload your review as an attachment if it exceeds 20,000 characters)

Reviewer #1: All comments have been addressed adequately by the authors. Accordingly I accept it for publication.Good luck

Reviewer #2: Dear Author

This revised version in good and acceptable, response requested comments point by point.

Reviewer #3: The authors adequately responded to all comments and performed all required modifications.

**********

7. PLOS authors have the option to publish the peer review history of their article (what does this mean?). If published, this will include your full peer review and any attached files.

If you choose “no”, your identity will remain anonymous but your review may still be made public.

**Do you want your identity to be public for this peer review?**  For information about this choice, including consent withdrawal, please see our Privacy Policy.

Reviewer #1: No

Reviewer #2: No

Reviewer #3: No

**********

# Editor report: Acceptance letter

**DOI:** 10.1371/journal.pone.0339270.r004

**Authors:** Lee Seong, Lee Seong

PONE-D-25-52195R1

PLOS One

Dear Dr. Hassaan,

I'm pleased to inform you that your manuscript has been deemed suitable for publication in PLOS One. Congratulations! Your manuscript is now being handed over to our production team.

At this stage, our production department will prepare your paper for publication. This includes ensuring the following:

* All references, tables, and figures are properly cited

* All relevant supporting information is included in the manuscript submission,

* There are no issues that prevent the paper from being properly typeset

You will receive further instructions from the production team, including instructions on how to review your proof when it is ready. Please keep in mind that we are working through a large volume of accepted articles, so please give us a few days to review your paper and let you know the next and final steps.

Lastly, if your institution or institutions have a press office, please let them know about your upcoming paper now to help maximize its impact. If they'll be preparing press materials, please inform our press team within the next 48 hours. Your manuscript will remain under strict press embargo until 2 pm Eastern Time on the date of publication. For more information, please contact onepress@plos.org.

You will receive an invoice from PLOS for your publication fee after your manuscript has reached the completed accept phase. If you receive an email requesting payment before acceptance or for any other service, this may be a phishing scheme. Learn how to identify phishing emails and protect your accounts at https://explore.plos.org/phishing.

If we can help with anything else, please email us at customercare@plos.org.

Thank you for submitting your work to PLOS ONE and supporting open access.

Kind regards,

PLOS ONE Editorial Office Staff

on behalf of

Dr. Lee Seong

Academic Editor

PLOS One
